# Practical Management of Adult Ultra-Rare Primary Retroperitoneal Soft Tissue Sarcoma: A Focus on Perivascular Epithelioid Tumours and Extraosseous Ewing Sarcoma

**DOI:** 10.3390/curroncol30070445

**Published:** 2023-06-21

**Authors:** Sameer S. Apte, Eyal Mor, Catherine Mitchell, David E. Gyorki

**Affiliations:** 1 Division of Cancer Surgery, Peter MacCallum Cancer Centre, Melbourne, VIC 3000, Australia; 2 Division of General Surgery, The Ottawa Hospital, Ottawa, ON K1H 8L6, Canada; 3 Division of Pathology, Peter MacCallum Cancer Centre, Melbourne, VIC 3000, Australia

**Keywords:** ultra-rare sarcoma, retroperitoneal sarcoma, perivascular epithelioid tumour, extraosseous Ewing sarcoma, extraosseous osteosarcoma, adult rhabdomyosarcoma

## Abstract

With the exception of well-differentiated liposarcoma, dedifferentiated liposarcoma, leiomyosarcoma, solitary fibrous tumour, malignant peripheral nerve sheath tumour, and undifferentiated pleomorphic sarcoma, the majority of the ≈70 histologic subtypes of retroperitoneal sarcoma are defined as ‘ultra-rare’ sarcomas, with an incidence of ≤1–5/1,000,000 persons/year. For most of these ultra-rare RPS subtypes, diagnosis and treatment follows international guidelines for the management of more common RPS histologies, with en bloc surgical resection as the mainstay of curative treatment, and enrolment in clinical trials where possible. Because the treatment of RPS is heavily driven by histology, the surgeon must be familiar with specific issues related to the diagnosis and management of ultra-rare sarcoma subtypes. Expert radiological and surgeon reviews are required to differentiate similarly presenting tumours where surgery can be avoided (e.g., angiomyolipoma), or where upfront systemic therapy is indicated (e.g., extraosseous Ewing’s sarcoma). Thus, the management of all retroperitoneal sarcomas should occur at a sarcoma referral centre, with a multidisciplinary team of experts dedicated to the surgical and medical management of these rare tumours. In this focused review, we highlight how diagnosis and management of the ultra-rare primary RPS histologies of malignant perivascular epithelioid cell tumour (PEComa), extraosseous Ewing sarcoma (EES), extraosseous osteosarcoma (EOS), and rhabdomyosarcoma (RMS) critically diverge from the management of more common RPS subtypes.

## 1. Introduction

### 1.1. Epidemiology of ‘Ultra-Rare’ Primary Retroperitoneal Sarcoma

Primary retroperitoneal sarcomas (RPS) are a heterogenous group of approximately 70 histologically and clinically distinct tumours, arising from the retroperitoneal fat, muscle, nerves, connective tissue, or blood vessels [1]. With an incidence of ≤1/100,000 persons/year, all primary RPS are defined as ‘rare cancers’ by the European ‘RARECARE’ and Canadian working groups on uncommon tumours [2,3]. Amongst all retroperitoneal tumours, the majority are well-differentiated or dedifferentiated liposarcoma (LPS, 62.8%), leiomyosarcoma (LMS, 19.3%), solitary fibrous tumour (SFT, 5.9%), malignant peripheral nerve sheath tumour (MPNST, 3.3%) and undifferentiated pleomorphic sarcoma (UPS, 2.2%) [4]. The remaining 6.6% of tumours, compromising the majority of the ≈70 distinct subtypes are each exceedingly rare with an incidence of ≤1–5/1,000,000 persons/year. Consequently, these histologies have been defined as ‘ultra-rare’ sarcomas by the Connective Tissue Oncology Society, deserving special attention [5]. A selection of the ultra-rare RPS histologies are listed below (Table 1). These histologies include extra-skeletal sarcomas (e.g., chondro-, osteo-, and Ewing sarcomas), Ewing-like sarcomas (e.g., desmoplastic small round cell tumours, *CIC*-rearranged, and *BCOR*-rearranged sarcoma), perivascular epithelioid cell tumours (PEComas), clear cell sarcomas, epithelioid sarcomas, angiosarcomas, fibrosarcomas, inflammatory myofibroblastic tumours, and dendritic cell sarcomas, amongst many others [Table 1].

### 1.2. Histology, Molecular Biology, and Genetics Drive Decision-Making for Retroperitoneal Sarcoma

Ultra-rare RPS, like more common RPS, usually only cause symptoms due to mass effect, and thus can grow unnoticed over long spans of time, reaching sizes of ≥10–20 kg. The insidious nature of RPS means that tumours are often discovered incidentally, or after presentation with non-specific complaints (e.g., increase in abdominal girth, early satiety, constipation, signs of venous compression, fatigue, weight loss, or B-symptoms). For many patients, ultra-rare RPS are already involving adjacent organs on presentation. Thus, en bloc compartmental surgical resection of associated organs (with or without neo- or adjuvant therapies) by an experienced sarcoma team remains the mainstay of curative-intent treatment for RPS. Consequently, an expert radiological and surgical review after appropriate cross-sectional imaging and biopsy is critical to delineate often highly distorted anatomic relations and biology of RPS [29].

Importantly, the prognosis and decision-making for RPS treatment is heavily driven by histology, and this is no different for ultra-rare RPS [30]. As a result, a percutaneous retroperitoneal coaxial core-needle biopsy with expert pathological review is required, with few exceptions [31,32]. As an example, for more common RPS subtypes, a TARPSWG review of 1007 patients found dramatic differences in local recurrence, overall survival, and the value of treatment strategy based on RPS histology [4]. Furthermore, the recently published STRASS phase III randomised trial, comparing neoadjuvant radiotherapy and upfront surgery for primary RPS, suggested some very important histology-specific differences: although a difference in abdominal recurrence-free survival across all histologies was not detected, a post hoc analysis found that neoadjuvant radiotherapy for patients with LPS histology showed a HR 0.62 with 95% CI [0.38, 1.02] that just barely missed the threshold for rejecting the null hypothesis [33]. Subsequently, the mixed-methods STREXIT study, which combined the STRASS cohort with STRASS-eligible patients who were not enrolled, did detect a difference in abdominal recurrence-free survival for the LPS subgroup, with the same effect estimate as STRASS (HR 0.61, 95% CI [0.42, 0.89]) [34]. These results suggest that the effect of radiotherapy for RPS is histology-specific, likely favouring the LPS subtype. Accordingly, although data is much sparser, the prognosis and management of ultra-rare sarcomas is dependent on histologic subtype, following the trends uncovered in the STRASS trial [5,12,35].

Due to advances over the last 30 years, molecular and genetic profiling of sarcomas now plays an increasingly important role in the accurate classification, prognosis, and treatment of ultra-rare RPS. In general, classifications of sarcomas based on molecular and genetic profiles fall into two main categories: those with a single genetic or molecular alteration, and those with complex alterations resulting in similar clinicopathologic phenotypes [36]. For instance, it is well known that the Ewing sarcoma is characterized by fusion to a single gene, *EWSR1*, while *MDM2* amplification by fluorescence in situ hybridization is present in nearly all LPS [37,38]. Some categories historically based on histology have been redefined by genetic and molecular profiling. For example, the non-Ewing round cell sarcoma is now known to be at least four distinct tumours based on various gene fusions: desmoplastic small round cell, non-ETS *EWSR1*, *CIC*-rearranged, and *BCOR* altered sarcomas [Table 1]. Moreover, molecular and genetic profiling of ultra-rare RPS has revealed some therapeutic targets for subtypes with characteristic driver mutations (e.g., *mTOR* inhibitors for *TSC*-mutated PEComa, and Tropomyosin kinase-receptor therapy for *NTRK*-fusion sarcomas). Finally, for sarcomas with complex alterations or no known driver mutations, next-generation or parallel array genomic sequencing may identify non-characteristic targetable mutations, allowing patient-specific tailored therapies to be used [39].

Because of the histologic, genetic, and clinical complexities related to the diagnosis and treatment of RPS and ultra-rare RPS, these patients should be managed only at a recognised multidisciplinary sarcoma centre [29]. Unsurprisingly, a United States National Cancer Database analysis found improved overall survival was associated with an annual volume of ≥13 RPS resections per centre. This key finding is likely explained by not only surgical expertise, but also the critical importance of an expert multidisciplinary team that tailors treatment to tumour biology and each individual patient. Consequently, the Transatlantic Australasian Retroperitoneal Sarcoma Working (TARPSWG) expert consensus has recommended a volume of 10–20 RPS resections yearly per centre as the minimum standard for a sarcoma referral centre [29].

### 1.3. The Challenge of Evidence-Based Medicine for Ultra-Rare Primary Retroperitoneal Sarcoma

As exemplified by the STRASS trial, even for the common histologies (e.g., LPS and LMS), the generation of practice-changing level I evidence has required extraordinary international collaboration, through initiatives like the Transatlantic Australasian Retroperitoneal Sarcoma Working Group [40]. For instance, the ongoing ‘STRASS-2’ phase III randomised trial investigating neoadjuvant chemotherapy for RPS will require massive international effort, but can still only focus on the more common LPS and LMS histologies, in order to maximize patient impact and maintain study power [33,41]. For the ‘ultra-rare’ RPS histologies, not only is randomised evidence effectively impossible to produce, high-quality data of any kind is also extremely challenging to obtain. Consequently, treatment for resectable ultra-rare primary RPS is based largely on subtype-specific studies that include non-retroperitoneal locations, as well as inferred from the management of the more common RPS histologic subtypes. TARPSWG has begun to address this knowledge gap through massive multi-centre retrospective and prospective registries of RPS, like the Retroperitoneal Sarcoma Registry (RESAR) [40]. Such efforts may eventually yield large enough analysable retrospective cohorts of ultra-rare primary RPS to help clinicians further refine management strategies. Moreover, advances in genomic sequencing and molecular profiling has prompted studies to include patients on a mutational basis, irrespective of histology. Consequently, a genomic or molecular approach to generating evidence for ultra-rare sarcoma may allow greater enrolment and higher study power [42].

Here, we highlight specific issues regarding some ultra-rare primary RPS histologies, including malignant PEComas, extraosseous Ewing sarcomas (EES), extraosseous osteosarcomas (EOS), and rhabdomyosarcomas (RMS). For these histologies, diagnosis or treatment critically diverges from the management of more common RPS subtypes. For the remainder of ultra-rare RPS histologies, diagnosis and treatment follow international guidelines for the management of RPS, with a focus on en bloc surgical resection as the mainstay of curative treatment. In general, due to the rarity of all RPS, patients should be offered enrolment in clinical trials. For ultra-rare RPS subtypes, clinical trials of novel therapies targeted to characteristic genetic abnormalities should be strongly considered. For all patients, referral should be made to a multidisciplinary sarcoma centre [29].

## 2. Perivascular Epithelioid Family Tumours of the Retroperitoneum

### 2.1. Overview

Perivascular epithelioid cell tumours (PEComa) are a rare family of mesenchymal tumours first described by Bonetti et al. in 1992 [43,44]. The hallmark of PEComas is the presence of histologically and immunohistochemically distinctive perivascular epithelioid (PEC) cells, for which there is no known normal tissue counterpart [1,45]. PEComas are characterised by expression of melanocytic markers (HMB45/Melan-A), cathepsin K, and the muscle differentiation markers actin and desmin [45,46]. Because PEComas demonstrate variable histology, and can occur at a wide variety of parenchymal, cutaneous, and soft tissue sites, no unifying description existed prior to Bonetti’s work. Instead, there was a disparate array of named entities, including the benign and malignant counterparts of pulmonary clear-cell sugar tumours (CCST), clear-cell myomelanocytic tumours (CCMT), angiomyolipomas (AML), and lymphangioleiomyomatosis (LAM). These tumours are now all known to be members of the same ‘PEComa-family’ of tumours, along with ‘PEComa-NOS’, which describes PEComas that are not easily categorised into these subtypes [45,47,48].

Primary retroperitoneal PEComa is ultra-rare, with less than 100 cases reported in the literature [49]. AML has a 4:1 preponderance for females, and targets young adults [45]. Similarly, LAM is almost exclusive to premenopausal women [50,51]. PEComa-NOS and epithelioid-variant AML have also demonstrated a clear association with female sex [52,53,54,55,56]. While the PEComa-family tumours can occur anywhere in the body, the retroperitoneal and abdominopelvic regions are most often affected by AML, LAM and PEComa-NOS, and less commonly CCST and CCMT [49,56,57,58]. AML is most frequently encountered in the renal parenchyma and perinephric fat, and less so in the liver, uterus, gastrointestinal tract, and retroperitoneum [59,60]. PEComa-NOS tumours are also often associated with the renal parenchyma and perinephric fat, but present at a higher variety of sites, including the retroperitoneum, abdominal wall, uterus, gastrointestinal tract, or any other viscera (commonly liver, pancreas, and skin) [56]. One notable variant, sclerosing PEComa-NOS, has a predilection for the perinephric retroperitoneum of middle-aged women, but has not yet been associated with more, or less, aggressive biology [45]. LAM is a tumour of the lymphatic system, most often causing a multifocal debilitating disease of the small airways and blood vessels [50,51]. Less commonly, it can form uterine tumours, gastrointestinal tumours, or as a primary lesion within the retroperitoneal lymphatics, where it tends to form a well-circumscribed single retroperitoneal mass [50,61].

### 2.2. Prognosis of Perivascular Epithelioid Tumours

While most PEComa-family tumours tend to be benign, causing local non-progressive disease (e.g., most AML, most LAM, and some PEComa-NOS), approximately 40–60% of PEComa are of uncertain malignant potential, or are frankly malignant [55,62]. Because the aggressiveness of PEComa is difficult to determine preoperatively, deciding the optimal management is challenging. For instance, although necrosis, haemorrhage, and calcification are more common in malignant retroperitoneal PEComas on imaging, both benign and malignant PEComas can also be well circumscribed and homogeneous, without obvious invasion [63]. Moreover, biopsies suggesting the epithelioid variant of AML were historically considered benign [52,53]. Subsequently, a portion of these monotypic epithelioid AML presented with lymph node or visceral metastases, despite bland primary histology [52,64]. These epithelioid AML tumours are now considered part of the potentially malignant PEComa-NOS spectrum [56]. Similarly, there have been reports of other PEComa-family tumours of the retroperitoneum with no high-risk features on primary pathology that eventually progressed with distant metastases [57].

To better categorize these tumours, Hornick et al. reported that aggressive PEComas usually show one or two of coagulative necrosis, lymphovascular invasion, mitotic figures, pleomorphic nucleoli and, rarely, lymph node metastases [45]. Later, Folpe et al. proposed that PEComa be categorized into ‘benign’, ‘uncertain malignant potential’, and ‘malignant’, based on the presence of two high risk features, including size > 5 cm, mitotic index > 1/5 mm^2^, high cellularity, infiltrative growth pattern, nuclear pleiomorphism, necrosis, and lymphovascular invasion [55,58]. Bleeker et al. proposed simplifying these criteria by considering only mitotic rate and size in a literature review of 234 PEComa-NOS cases (54 retroperitoneal/abdominopelvic) [56] [Table 2]. In Bleeker’s review, they evaluated both grading systems, finding no metastases or aggressive behaviour in PEComas classified as benign by Folpe’s criteria. However, of all tumours that eventually metastasized or recurred (30%), only 51% were classified as malignant, with the remainder classified as ‘uncertain malignant potential’. Importantly, there were no recurrences for tumours ≤ 5 cm without other high-risk features, highlighting the indolent nature of this specific subset. Finally, size > 5 cm and mitotic rate > 1/mm^2^ were the only factors found to be associated with recurrence after curative-intent resection (HR = 4.30, 95% CI [1.23, 27.14] *p* = 0.02, and HR = 7.56, 95% CI [2.97–23.19] *p* < 0.001, respectively) [56]. More recently, in perhaps the most complete analysis of advanced PEComas, a systematic review of 124 PEComa-NOS cases by Bourgmayer et al. found that only risk category and presence of metastases were independently associated with overall survival on multivariable analysis (HR = 4.66, 95% CI [1.07, 20.19] *p* = 0.039, and HR = 2.59, 95% CI [1.06, 6.33] *p* = 0.036, respectively) [55]. Finally, of patients who presented with PEComa metastases, only lymph node involvement was independently associated with OS (HR = 3.11, 95% CI [1.13, 8.60] *p* < 0.05). Importantly, Bourgmayer’s analysis did not have sufficient study power to evaluate PEComas treated with curative-intent surgery. While Folpe’s and Bleeker’s criteria are reasonable strategies for prognostication of retroperitoneal PEComa, given the limited available data considerable uncertainty remains [55,56,58,65].

For malignant PEComas specifically arising from the retroperitoneum or perinephric area, Touloumis et al. conducted the only systematic review of case reports, estimating a malignancy rate 60% and 44%, respectively [49]. Importantly, nearly half of PEComas categorized as malignant either presented with or developed metastases. Consequently, retroperitoneal PEComa tumours that are extra-visceral, and not diagnosed as classical AML, likely have a substantially higher rate of aggressive behaviour. Radical surgical resection is usually required. Common sites of metastases include the lungs, liver and bone, although metastases to brain, skin and the abdominopelvic region have been described [55,56,62,66,67]. Importantly, PEComas may metastasize up to 10 years after initial curative-intent resection [66,68,69]. Thus, patients with resected tumours should undergo long-term surveillance similar to other RPS.

### 2.3. Differentiating Malignant PEComa-NOS from Benign AML

**Figure 1:**
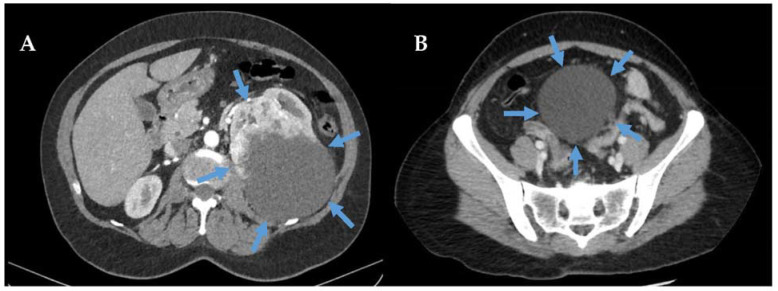
Primary retroperitoneal benign, AML arising from the kidney (blue arrows, Panel (**A**)) and primary retroperitoneal malignant PEComa-NOS arising from the rectosigmoid mesentery (blue arrows, Panel (**B**)). Both show similar CT characteristics, including density inconsistent with fat (−52 and −39 Hounsfield units, respectively) and well-circumscribed borders apart from the organ of origin (Kidney in Panel (**A**), and sigmoid in Panel (**B**)). While both AML and PEComa commonly arise from the kidney and perinephric fat, AML rarely, if ever, arises directly from the retroperitoneal tissues. Retroperitoneal core biopsy confirmed the diagnosis in both cases. The AML was managed with preoperative angioembolization and marginal excision of the left kidney and retroperitoneal tumour en bloc (R0 resection). The malignant PEComa-NOS was managed with radical resection, including rectosigmoid and low anterior resection, clearance of the retroperitoneal tissues above the aortoiliac axis, and right hemi-colectomy due to cecal abutment (R0 resection).

While significant uncertainty remains in determining if some PEComa-family tumours are malignant or not, one issue of considerable importance is accurately diagnosing benign renal AML. Benign renal AML can often be confidently diagnosed on magnetic resonance imaging or computed tomography without the need for biopsy [70]. It is characterised by a copious amount of intratumoral fat density, a renal parenchymal defect, encapsulated margins, and enlarged/aneurysmal intralesional blood vessels [71]. Radiologic reviews of retroperitoneal tumours where benign AML is a consideration should be performed at a sarcoma referral centre, as AML can look similar to well-differentiated or dedifferentiated LPS or PEComa-NOS on imaging [Figure 1] [71,72,73]. This is due to the scarce or absent intratumoral fat of benign epithelioid AML on imaging. Thus, tumours that cannot clearly be diagnosed as AML or PEComa-NOS on imaging require retroperitoneal coaxial core-needle biopsy for diagnosis [70]. Similarly, some LPS (especially well-differentiated LPS) may look similar to AML, necessitating biopsy and testing for fluorescence in situ hybridization amplification of the *MDM2* and *CDK4* genes for LPS diagnosis [71,73,74]. Importantly, even on biopsy, epithelioid AML resembles renal cell carcinoma, although immunohistochemistry can usually distinguish these entities. Thus, all cases and pathologic specimens should be reviewed at a multidisciplinary sarcoma centre [70].

Importantly, if benign AML is diagnosed, radical surgery can often be avoided. The management of benign renal AML is not the focus of this review, but often includes selective arterial embolization, surveillance, marginal surgical resection with nephrectomy, or possibly partial nephrectomy. Choice of treatment depends on the risk of malignancy or haemorrhage, which are suggested by size > 6 cm, growth rate > 2.5 mm/year, presence of aneurysmal vessels > 5 mm, epithelioid features, or aggressive histology [75]. The remainder of cases (PEComas of either malignant, or of uncertain malignant potential) should be managed similar to other RPS, with en bloc compartmental resection of involved organs with or without adjuvant therapies as standard-of-care.

### 2.4. Genetics and Systemic Therapies Specific to PEComa-NOS

In general, PEComa tumours display inappropriate activation of the mammalian target of rapamycin (*mTOR*) pathway [69,76,77]. Common genetic alterations that lead to *mTOR* activation include *TSC1/2* mutations, *TFE3* gene fusions, and *FLCN* mutations [76,78]. *TSC1/TSC2* gene mutations define the autosomal dominant genetic disorder ‘tuberous sclerosis’ (TS) which is characterised by dermatologic changes (e.g., hypopigmented macules or ‘ash-leaf’ spots), epilepsy, cognitive and behavioural deficits, benign brain lesions, wide-spread hamartomatous tumours, renal/perinephric AML, and LAM [79,80]. In TS patients, PEComa-family tumours tend to be multiple, bilateral, and benign. While TS does occasionally result in malignant PEComa-NOS (often renal or perinephric), PEComa-NOS is usually sporadic [51,79]. PEComa tumours are known to be chemotherapy resistant. In a case series of 53 patients by Sanfilippo et al., anthracycline- and gemcitabine-based regimens showed poor median progression free-survival (PFS) of 3.2 and 3.4 months, and low response rates of 13% and 20%, respectively [56,81,82]. Because activation of the *mTOR* pathway is implicated in PEComa, *mTOR* inhibitors (sirolimus, everolimus, and tasirolimus) are often the first-line treatment for metastatic PEComa, with somewhat better PFS of 9 months and response rates of 40% in case series [81,83,84,85]. Recently, significant collaborative effort has led to the publication of the AMPECT study, the first prospective clinical trial in this ultra-rare tumour group [86]. This trial investigated nab-sirolimus, a nanoparticle-albumin bound formulation of sirolimus in patients with metastatic, unresectable PEComas of any anatomic site, who had not previously received an *mTOR* inhibitor. Of note, 25/34 patients in the trial had a PEComa in a retroperitoneal location or organ. At 6-month follow-up, the overall response rate was 39% (95% CI [22, 58]) and PFS, 10.6 months (95% CI [55, -]), results which are consistent with previous case series [55,81,86]. AMPECT also confirmed increased activity of nab-sirolimus in *TSC2* mutated patients (89% response), as compared to other mechanisms of *mTOR* pathway activation (13% response, *p* < 0.001). Importantly, at median follow-ups of 2.5 years, 7 out of 12 patients who had previously responded to nab-sirolimus were still receiving treatment, indicating durable response is possible in some patients.

## 3. Extraosseous Ewing Sarcoma of the Retroperitoneum

### 3.1. Overview

Extraosseous Ewing sarcoma (EES) and intraosseous Ewing sarcoma (IES) are aggressive tumours that belong to the Ewing sarcoma family of tumours (ESFT) [87]. These sarcomas share a common neuroectodermal histology and genetic profile [37,87,88,89,90,91,92]. Originally, IES, EES, and primitive neuroectodermal tumour (PNET) were thought to be distinct tumours. For instance, ‘Homer Wright’ rosettes of oval nuclei around a fibrous core identified on histology are characteristic of PNET, but not IES or EES [93,94]. Discovery that the vast majority of ESFT are defined by the same chromosomal translocations involving the *EWSR* gene (e.g., t(11;22) (q24;q12) or t(21;22) (q22;q12)) confirmed a common genetic origin of these tumours, suggesting that PNET is a more differentiated version of classical ESFT [87]. It is now known that 95% of ESFT harbour a characteristic *EWSR1-FLI1* fusion gene (85%) or less commonly *EWSR1-ERG* (5–10%) [87,95,96] that can be detected by fluorescence in situ hybridization (FISH) or reverse-transcriptase polymerase chain reaction (RT-PCR) [37,92]. Interestingly, rarer fusion variants, such as *EWSR1-FUS/FEV*, may have a higher predilection for extraosseous and retroperitoneal sites [97].

EES is an ultra-rare sarcoma with an annual incidence is about 0.4/million persons. Retroperitoneal EES comprises ≈20–30% of those cases [98,99]. Due to its rarity, only case reports and small case series exist for retroperitoneal EES [100,101,102,103,104,105,106,107]. Management of retroperitoneal EES is inferred from studies on all Ewing sarcomas or EESs that included non-retroperitoneal sites. While IES tends to present in teenage or young adult males with a median age of 25 years, EES has less of a gender bias (45.2% females vs. 54.8% males), and has a bimodal age distribution with higher incidence in patients ≤ 5 years old or patients ≥ 30 years old [99,108,109]. Common retroperitoneal and abdominopelvic sites of ESS include the kidney, retroperitoneum, gastrointestinal tract and uterus [99,101,109,110,111,112,113]. Interestingly, Applebaum et al. found that PNET histology is more common in EES (including retroperitoneum) as compared to IES (59.3% of EES had PNET histology vs. 6.4% of IES, *p* < 0.001), a finding confirmed by Lynch et al. in an analysis of 978 EES patients (25.5% of EESs had PNET histology vs. 2.4% of IES) [99,109]. Like more common RPS subtypes, symptoms of retroperitoneal EESs tend to be minimal or vague, allowing insidious tumour growth to large sizes where multiple organ invasion has often already occurred.

### 3.2. Prognosis of Retroperitoneal Extraosseous Ewing Sarcoma

Unlike retroperitoneal PEComa, which can sometimes be indolent or benign, retroperitoneal EES is an aggressive, high-grade tumour that, if left unimpeded, will grow and metastasize widely. Thus, approximately 30–40% of patients with EES present with metastases at diagnosis [99,109]. The most common sites of metastatic disease are the lung (80% of metastases) and bone marrow (10%) [114,115,116]. Brain, visceral, lymph node, and soft tissue metastases are also reported [114,115,116]. EES and IES have similar overall survival [99,109]. These findings are corroborated by other smaller case series published previously [117,118]. However, when considering patients who present with localized disease, EES may have a slightly better prognosis as compared to IES, as reported by Applebaum et al. (10-year OS = 65.2% 95% CI [59.3, 70.5%] for localized EES vs. 55.3% 95% CI [51.5, 58.8] for localized IES) [99]. Survival for the cohort of metastatic EES and IES is poor, even with multimodal therapy, and was not different between groups (median survival IES = 47.5 months 95% CI [42.2, 55.1] vs. EES = 48.2 months 95% CI [40.2, 60.7], *p* = 0.82) [109]. For retroperitoneal EES, it is important to note that PNET histology is not only more common but may have a worse prognosis than classical EES. For example, Schmidt et al. found worse overall survival at 7.5 years EES with PNET histology versus classical EES (45% vs. 67%, *p* = 0.026). Moreover, Liu et al. reviewed 161 PNETs (46 of which were abdominopelvic) showing a 5-year overall survival of 54% with modern multimodality treatment [113]. Finally, in a review of over 2668 Ewing sarcoma patients, PNET histology was associated with worse OS for EES patients (HR 1.33, 95% CI [1.04, 1.70] *p* = 0.02) [109]. Aside from histology, other poor prognostic factors for OS in EES are size ≥ 5–10 cm, presence of lymph node metastases, cancer stage, and use of multimodal therapy [109,113]. Due to its aggressive nature, newly diagnosed retroperitoneal EES/PNET should almost always be managed as a high-grade sarcoma.

### 3.3. Multimodal Therapy for Retroperitoneal Extraosseous Ewing Sarcoma

**Figure 2:**
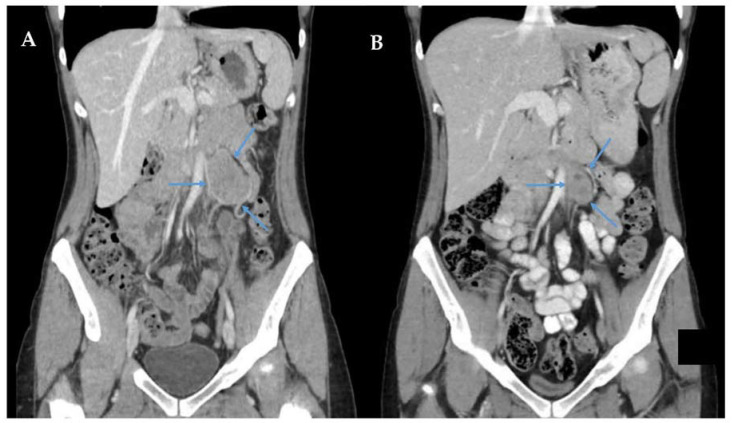
Primary retroperitoneal extraosseous Ewing sarcoma (blue arrows) before (Panel (**A**)) and after (Panel (**B**)) neoadjuvant VDC/IE, with 60% tumour volume reduction. This patient underwent R0 resection of RPS with en bloc resection of the anterior wall of the aorta and inferior mesenteric artery. Due to good response from chemotherapy, the left ureter, left kidney, and left colon were successfully preserved.

Due to the common genetic and cellular origins, ESFTs at all anatomic locations have similar tumour biology (including retroperitoneal EES) and, therefore, similar treatment principles [109,115,119]. Because EES and IES are both highly chemosensitive, the management of retroperitoneal EES critically diverges from standard RPS management by emphasizing the crucial role of dose-intensive chemotherapy [120]. Rud et al. published the first case series of chemotherapy in EES, noting that patients treated after 1970 (when chemotherapy for ESFT was popularized) had a 48% 5-year OS, versus 28% for those treated before 1970 [114]. Subsequently, dose intensive protocols became popular, with further improvements in overall survival. For instance, Kolb et al. observed a 4-year event-free survival rate of 82% with the modified ‘P6’ protocol of vincristine, doxorubicin, cyclophosphamide, ifosfamide, and etoposide (VDC/IE) [119]. Most recently, the landmark ‘EURO EWING 2012’ randomised trial in 640 ES patients showed that compressed dosing of VDC/IE (standard in North America) had a 99% probability of better event-free survival by Bayesian analysis (HR 0.71 95% CI [0.55–0.92]) as compared to VIDE (common in Europe) [115]. Moreover, VDC/IE was less toxic, had a shorter duration of treatment, and higher completion rates of therapy for both localised and metastatic ESFT than VIDE. Importantly, nearly all subgroups evaluated showed a magnitude of effect that likely favoured VDC/IE. Thus, for all ESFT, including retroperitoneal EES, initial treatment with VDC/IE and subsequent multi-visceral resection should now be considered standard of care [Figure 2] [115].

Surgery and radiation, however, are still critical to the management of EES and retroperitoneal EES. For instance, Lynch et al. investigated 978 EES and 1682 IES patients, observing that combination therapy with chemotherapy and local therapy (radiation or surgery) was strongly associated with survival for EES (HR 0.42, 95% CI [0.31, 0.55] *p* < 0.001), while surgery alone, radiation alone, or chemotherapy alone were not [109]. Additionally, other smaller case series, have confirmed the importance of multimodal therapy for EES, with a focus on chemotherapy followed by surgery ± radiation [91,93,107,121]. ESFTs are known to be radiosensitive, but the role of radiation has decreased in the past two decades, likely due to improvements in chemotherapeutic regimens [99]. In our centre, neo- or adjuvant radiation for retroperitoneal EES still plays an important role to help sterilize surgical margins, or to facilitate sparing critical structures that are abutting, but not invaded by tumour (e.g., the femoral nerve). Additionally, for patients who are poor surgical candidates, primary chemotherapy and radiation can be an option. Consequently, unlike more common RPS subtypes, initial therapy for non-metastatic retroperitoneal EES should be neoadjuvant VDC/IE followed by radical resection ± neoadjuvant or adjuvant radiation.

## 4. A Note about Extraosseous Osteosarcoma and Adult Rhabdomyosarcoma

### 4.1. Extraosseous Osteosarcoma

**Figure 3:**
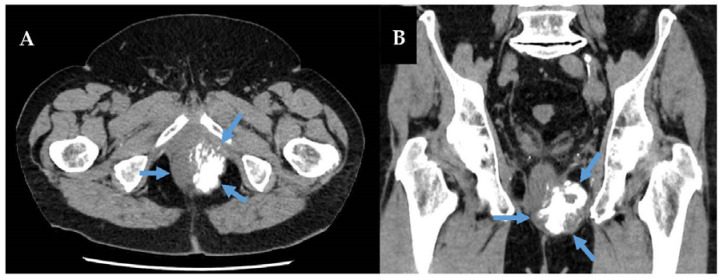
Primary retroperitoneal extraosseous osteosarcoma (blue arrows) axial (Panel (**A**)) and coronal (Panel (**B**)) views. This tumour shows CT characteristics including a deep-seated pelvic tumour with significant calcification matrix, and invasion into the rectum, prostate, pelvic floor muscles, and left pelvic sidewall. This patient underwent R0 resection of RPS with pelvic exenteration including en bloc extralevator abdominoperineal resection, cystoprostatectomy, clearance of ischiorectal fossa and ischial tuberosity without bony involvement.

In addition to extraosseous Ewing sarcomas, extraosseous osteosarcomas (EOS) and rhabdomyosarcomas (RMS) can rarely present in the retroperitoneum [6,122,123,124,125,126,127]. Importantly, in 5–10% of cases, de-differentiated LPS and 10–15% of malignant peripheral nerve sheath tumours can exhibit divergent differentiation with osteosarcomatous changes, mimicking primary or metastatic osteosarcoma [13,128,129,130]. Moreover, some high-grade osteosarcomas can also show amplification of *MDM2*, which is commonly used to confirm a diagnosis of WDLPS/DDLPS [131]. Usually, DDLPS with osteosarcomatous change will co-exist with a WDLPS component, and show amplification of *CDK4* in addition to *MDM2*, features not seen in primary osteosarcoma [38] [Figure 3]. On imaging, retroperitoneal EOS are often deep-seated tumours with a large, well-developed calcification matrix with heterogeneous, necrotic soft tissue tumour growth, and invasion into surrounding muscles and abdominopelvic organs. Conversely, osteosarcomatous DDLPS often shows coarse, but less well developed, calcifications, with surrounding neoplastic adipose tissue, and abdominopelvic organ invasion [132,133]. Unlike Ewing sarcoma, the management of primary retroperitoneal EOS follows accepted guidelines for RPS or primary soft-tissue tumours, and not intraosseous osteosarcoma [29,120]. For example, multimodal therapy with chemotherapy and surgery ± radiation has a well-established role in curative-intent treatment for IOS, with disease-free survival increasing from ≈20% to ≈60% with chemotherapy [120,134]. In contrast, the role of chemotherapy in localized EOS is controversial, as response rates are much poorer. For instance, Heng et al. reported retrospective results on 370 localized EOS cases from 24 sarcoma centres, finding no association of chemotherapy with decreased local or systemic recurrence [135]. Neo- or adjuvant radiation, however, was found to be associated with decreased local recurrence (HR = 0.46, 95% CI [0.26, 0.80] *p* = 0.01), but not survival. These findings are corroborated by other case series and a systematic review of EOS [136,137,138,139]. Accordingly, the ‘European Society of Medical Oncology’ EURO-Can guidelines note that primary EOS and IOS are likely biologically distinct entities. Thus, unlike IOS, EOS is treated as a primary soft-tissue tumour with radical resection as the mainstay of treatment [120,140].

### 4.2. Adult Rhabdomyosarcoma

In children, rhabdomyosarcoma (RMS) occurs at any anatomic location, and is the most common sarcoma, representing 5% of all paediatric cancers [141]. In contrast, RMS is an ultra-rare sarcoma in adults, with only a handful of reported primary retroperitoneal cases [142,143,144]. Similar to retroperitoneal osteosarcoma, a diagnosis of dedifferentiated liposarcoma with heterologous rhabdomyosarcomatous transformation should be considered and excluded [128]. Of reported retroperitoneal RMS, pleomorphic and alveolar subtypes are more commonly encountered, while embryonal and RMS-‘not otherwise specified’ are less common [145]. Importantly, it has been suggested that adult retroperitoneal rhabdomyosarcoma (RMS) may have distinct tumour biology compared to its paediatric counterpart. For instance, the chemo-resistant pleomorphic subtype may be more common in adults [146]. Additionally, adult RMS may have worse prognosis and poorer response to multimodal therapy as compared to paediatric RMS [141,147,148]. For instance, in a large analysis of 2600 patients in the ‘SEER’ database, 5-year OS was 27% ± 1.4 vs. 61% ± 1.4 for adult versus paediatric RMS, and this trend was consistent for localized disease (5-year OS 82% ± 2.0% for children and 47% ± 2.9% for adults, *p* < 0.001) [148]. Similarly, a single-centre study of 50 adults in Denmark with RMS showed a 5-year overall survival of 40% for localized disease and 15% for metastatic disease, well below outcomes for children [149]. Other studies have corroborated these poorer outcomes in adults [150]. In contrast, however, Ferrari et al. analysed 171 adult RMS patients (all anatomic locations) by scoring these cases based on their adherence to paediatric chemotherapy treatment protocols [151]. Using this approach, they found a response rate to chemotherapy of 85% with 5-year OS of 61% for those sarcomas treated according to paediatric principles. Additionally, Gerber et al. at Memorial Sloan Kettering Cancer Centre analysed 148 adult RMS patients, finding that those treated on a prospective chemotherapy protocol had better survival (5-year OS of 54% vs. 36%) [152]. A systematic review and pooled analysis of 553 adults with low- and intermediate-grade RMS corroborated these findings, showing decreased usage of chemotherapy to be an important risk factor for mortality [153]. Finally, a series of 82 adult RMS patients from MD Anderson Cancer Centre found that chemotherapy was only offered to 37% of patients as a part of multimodality therapy, and radiation alone was used 11% of the time [154]. Thus, poorer outcomes in adult RMS versus paediatric RMS may not be due to an inherent difference in tumour biology, but partially attributable to lower protocolized chemotherapy usage in adults [155]. While the role of chemotherapy in adult retroperitoneal RMS is controversial, radical resection plus neo- or adjuvant chemotherapy following paediatric treatment algorithms is an accepted approach with a potential survival benefit and is the preferred approach in our centre [156].

## 5. Conclusions and Future Directions

For the majority of ultra-rare RPS, diagnosis and management follow accepted international guidelines for more common RPS, with a focus on curative-intent resection as the mainstay of treatment. For some tumour types, including retroperitoneal PEComa and EES, clinicians should be aware of histology-specific caveats that greatly impact diagnosis and management. For malignant PEComa, it is critical to differentiate these tumours from benign PEComa-family tumours (like AML), where multi-visceral resection and biopsy can often be avoided. For retroperitoneal EES/PNET, neoadjuvant VDC/IE with en bloc resection of involved organs ± neo- or adjuvant radiation is the standard of care. Advances in further classifying ultra-rare RPS based on their genetic and molecular characteristics has prompted optimism in identifying targeted therapies to serve as an adjunct to multi-visceral resection. At present, enrolment in mutation-specific clinical trials of novel immuno- or molecular therapies based on next generation sequencing remain the best treatment option for patients with advanced disease. No matter the histology or genetic profile, all patients suspected to have RPS, whether ultra-rare or not, should be referred to a sarcoma reference centre for diagnosis and definitive management. Further research to better characterize the natural history and improve clinical outcomes for ultra-rare RPS will be dependent on massive international collaboration, like that of the Transatlantic Australasian Retroperitoneal Sarcoma Working Group [40].

## Figures and Tables

**Table 1:** A Selection of ‘Ultra-Rare’ Sarcoma Subtypes Occurring in the Retroperitoneum.

	Common Genetic or Molecular Findings	* Special Management Considerations
**PEComa-family tumours**		
Angiomyolipoma	*TSC1/2* or *FLCN* deletions, *SFPQ/DVL2/NONO*::*TFE3*	Embolization, marginal resection, or surveillance
PEComa-NOS/CCMMT/CCST	*TSC1/2* or *FLCN* deletions, *SFPQ/DVL2/NONO*::*TFE3*	* *mTOR* inhibitors may have role in *TSC* mutated patients
**Extraosseus and classic Orthopaedic** **sarcoma**		
Ewing sarcoma	*EWSR1*::*FLI1/ERG/FUS/FEV/ETV/E1AF*	Neo- or adjuvant VDC/IE
Osteosarcoma	FISH amplification of *MDM2*, but not *CDK4* (unlike LPS)	* Treat similar to RPS, unlike intraosseous osteosarcoma
Myxoid chondrosarcoma [6]	*NR4A3*::*EWSR1/TAF1*	* sunitinib/pazopanib may have role (phase II trials)
Synovial sarcoma [7]	*SS18*::*SSX1/2*	*
Rhabdomyosarcoma [8]	*PAX3/7* fusions. Alveolar: *FKHR/NCOA1*, spindle-cell: (*NCOA2*), embryonal: (complex alterations)	Neo- or adjuvant chemo (paediatric protocols). Pleomorphic subtype: chemoresistant
**Other non-Ewing round cell** **RPS** [9]		
EWSR1 non-ETS sarcoma	*EWSR1*::*PATZ1*	*
CIC-rearranged sarcoma	*CIC*::*DUX4*	*
BCOR-altered sarcoma	*BCOR*::*CCNB3*	*
Desmoplastic small round cell tumour	*EWSR1*::*WT1*	* Potential role for CRS-HIPEC
**Other ultra-rare RPS histologies**		
Epithelioid sarcoma [10]	*SMARCB1/INI1* mutations	* Consider resecting associated lymph node basins
Clear cell sarcoma [11]	*EWSR1*::*ATF1/CREB1*	*
Angiosarcoma [12]	Complex alterations (secondary angiosarcoma: *MYC* amplification)	*
Inflammatory myofibroblastic tumour [13]	*TPM3/4/CLTC*::*ALK*	*
NTRK-fusion sarcomas [14,15]	*NTRK3*::*ETV6/EML4*	* Tropomyosin kinase-receptor therapy has a role
Myxoinflammatory fibroblastic cell tumour [16]	Complex alterations *TGFBR3, VGLL3, BRAF*	*
Myofibroblastic sarcoma [17]	Complex or unknown alterations	*
Fibrosarcoma [18]	Complex alterations, *CDK2NA/B, TP53, EWSR1*	*
Myxofibrosarcoma [19,20]	Complex alterations, *CDK2NA/B, TP53, RB1*	*
Low grade fibromyxoid sarcoma [21]	*FUS*::*CREB3L2*	*
Alveolar soft part sarcoma [22]	*ASPSCR1*::*TFE3*	*
Extrarenal malignant rhabdoid tumour [23]	*SMARCB1* inactivation	*
Epithelioid hemangioendothelioma [24]	*WWTR1*::*CAMTA1, YAP1*::*TFE3*	*
Angiomatoid Fibrous Histiocytoma [25]	*EWSR1*::*CREB1/ATF1, FUS*::*ATF1*	*
Intimal Sarcoma [26]	Complex alterations, notably *MDM2/CDK4* amplification. Additionally, *PDGFRA/B*, *NOTCH2, CDKN2A/B*	*
Malignant myoepithelioma [27,28]	Complex alterations, *EWSR1* rearrangements	*

* Management of these ultra-rare subtypes follows accepted international guidelines for retroperitoneal sarcomas, with en bloc resection as the standard of care. PEComa-NOS—perivascular epithelioid cell tumour, not otherwise specified. CCMMT—clear cell myomelanocytic tumour, CCST—clear cell sugar tumour, VDC/IE—vincristine, doxorubicin, cyclophosphamide, ifosfamide, etoposide, LPS—liposarcoma, FISH—fluorescence in situ hybridization, RPS—retroperitoneal sarcoma, CRS-HIPEC—cytoreductive surgery and heated intraperitoneal chemotherapy.

**Table 2:** Proposed Criteria to Stratify PEComa-NOS Tumours into Benign, Malignant, and Uncertain Malignant Potential.

	Benign	Malignant	Uncertain Malignant Potential
**High-risk features**			
**Folpe** [31]			
Size > 5 cm Mitotic rate ≥ 1/50 HPF Infiltrative growth pattern High nuclear grade and cellularity Necrosis Vascular invasion	<2 high-risk features and size ≤ 5 cm	2 or more of any high-risk features	(1) Size > 5 cm with no other high-risk features, or (2) only high nuclear grade/multi-nucleated giant cells
**Bleeker** [29]			
Size > 5 cm Mitotic rate ≥ 1/50 HPF	No high-risk features	Both high-risk features	Only one high-risk feature

PEComa—perivascular epithelioid cell tumour, NOS—not otherwise specified, HPF—high power field.

## Data Availability

Not applicable.
